# Differential effects of EPA versus DHA on postprandial vascular function and the plasma oxylipin profile in men[S]

**DOI:** 10.1194/jlr.M067801

**Published:** 2016-09

**Authors:** Seán McManus, Noemi Tejera, Khader Awwad, David Vauzour, Neil Rigby, Ingrid Fleming, Aedin Cassidy, Anne Marie Minihane

**Affiliations:** Department of Nutrition and Preventive Medicine, Norwich Medical School,* University of East Anglia, Norwich NR4 7UQ, United Kingdom; Institute for Vascular Signalling, Centre for Molecular Medicine,† Goethe University, 60590 Frankfurt, Germany; Institute of Food Research,§ Norwich NR4 7UA, United Kingdom

**Keywords:** augmentation index, blood pressure, fish oil, hydrogen sulfide, lipidomics, nitric oxide, nutrition, omega-3 fatty acids, pulse wave velocity, eicosapentaenoic acid, docosahexaenoic acid

## Abstract

Our objective was to investigate the impact of EPA versus DHA on arterial stiffness and reactivity and underlying mechanisms (with a focus on plasma oxylipins) in the postprandial state. In a three-arm crossover acute test meal trial, men (n = 26, 35–55 years) at increased CVD risk received a high-fat (42.4 g) test meal providing 4.16 g of EPA or DHA or control oil in random order. At 0 h and 4 h, blood samples were collected to quantify plasma fatty acids, long chain n-3 PUFA-derived oxylipins, nitrite and hydrogen sulfide, and serum lipids and glucose. Vascular function was assessed using blood pressure, reactive hyperemia index, pulse wave velocity, and augmentation index (AIx). The DHA-rich oil significantly reduced AIx by 13% (*P* = 0.047) with the decrease following EPA-rich oil intervention not reaching statistical significance. Both interventions increased EPA- and DHA-derived oxylipins in the acute postprandial state, with an (1.3-fold) increase in 19,20-dihydroxydocosapentaenoic acid evident after DHA intervention (*P* < 0.001). In conclusion, a single dose of DHA significantly improved postprandial arterial stiffness as assessed by AIx, which if sustained would be associated with a significant decrease in CVD risk. The observed increases in oxylipins provide a mechanistic insight into the AIx effect.

An impact on arterial stiffness and compliance, associated with reduced blood pressure (1–4), is thought to be a significant contributor to the lower CVD risk associated with increased long chain n-3 PUFA (LC n-3 PUFA; EPA plus DHA) intake and status (4–6).

In randomized controlled trials (RCTs) conducted to date, vascular function is typically measured in the fasting nonchallenged state. Given that adults following Westernized dietary patterns typically spend 16–18 h per day in the postprandial state, and that a postprandial phenotype characterized by an exaggerated lipemia and glycemia and inflammatory and oxidative stress is associated with vascular dysfunction (7), the postprandial vascular response is likely to be a more significant and discriminating marker of CVD risk. However, the impact of dietary factors and meal composition on acute postprandial vascular function is poorly understood.

Increasing the total fat content of a test meal reduces both cardiac and peripheral postprandial vascular reactivity (7) and a small number of studies providing EPA plus DHA in the test meal have reported a positive effect of fish oil on vascular tone over 2–8 h (8–12). Although rarely considered, variability in the EPA and DHA ratio of the n-3 PUFA (fish or fish oil) source used in RCTs is likely to be a major determinant of the apparent inconsistency in findings reported.

A limited number of chronic interventions that measured fasting vascular function indicate that DHA has a greater beneficial effect than EPA (4). However, with the exception of a single trial that examined relative impact on the cardiovascular hemodynamic responses to exercise (11), no study has directly compared the effects of EPA- versus DHA-rich oils on the postprandial response.

Identification of the most bioactive LC n-3 PUFA or EPA/DHA ratio on “health” end points is important in order to refine current dietary recommendations and allow the stratification of intervention to suit an individual’s phenotype. Furthermore, with the emergence of nonfish sources of these fatty acids, such as separate EPA and DHA produced by marine microalga or in transgenic seed oils, and the availability of oil blends, all with a defined EPA and DHA content, information on their relative bioactivity is needed in order to inform these production systems (13, 14).

The primary aim of this double-blind, placebo-controlled acute test meal study was to compare the impact of EPA versus DHA on postprandial (4 h) vascular function as assessed by blood pressure (BP), pulse wave velocity (PWV), reactive hyperemia index (RHI), and augmentation index (AIx). Underlying molecular mechanisms were investigated by quantifying potential circulating mediators of vascular function including nitrite [as a biomarker of nitric oxide (NO)], hydrogen sulfide (H_2_S), and LC n-3 PUFA hydroxy, epoxide, and thiol metabolites. Although shown to be potent modulators of vascular tone in cell, animal, and human ex vivo studies (15, 16), the role of these compounds in modulating EPA- and DHA-induced changes in vascular function in humans is unknown.

## MATERIALS AND METHODS

This single-center dietary intervention study was conducted at the Norwich and Norfolk University Hospital Clinical Research Facility, at the University of East Anglia, between September 2012 and September 2013. All participants provided informed consent prior to commencing the study. It was conducted according to the guidelines described in the Declaration of Helsinki, and ethical clearance was provided by the English National Research Ethics Service, East of England Research Ethics Committee (reference 12/EE/0011). This trial was registered at www.clinicaltrials.gov (#NCT01692431).

### Subjects

Twenty-six males between the ages of 35 and 55 years were recruited through local and targeted online advertisement. Volunteers were recruited if they possessed one or more of the following CVD risk factors: total cholesterol (TC) ≥6 mM, HDL cholesterol (HDL-C) ≤1.0 mM, systolic BP (SBP) >140 mmHg, diastolic BP (DBP) >90 mmHg, or waist circumference >102 cm, which individually were associated with an aged-adjusted relative risk (RR) of CVD ≥1.5, as previously described by Wilson et al. (17). Exclusion criteria included any clinically diagnosed disease, BP ≥160/100 mmHg, abnormal liver function or blood cell count, use of lipid-lowering or insulin-sensitizing medication, use of steroids or nonsteroidal anti-inflammatory drugs including aspirin, antibiotics use, or vaccinations within the previous 3 months, use of dietary fish oil or high dose antioxidant vitamin supplements, habitual consumers of more than one portion (140 g) of oily fish per week, heavy drinkers [>30 units (240 g) of alcohol per week], smokers or ex-smokers ceasing <3 months before screening.

### Study protocol

On the three separate study days at least 4 weeks apart, meals containing EPA-rich oil (ERO), DHA-rich oil (DRO), or control oil (CO) were consumed. Treatment order for each participant was randomized by an independent researcher, who was not involved in the study design, data collection, or data analysis. For 3 days prior to their clinical visits, subjects were asked to follow a restricted diet that included avoidance of n-3 PUFA-rich foods and nitrate-, nitrite-, sulfite-, and sulfate-rich foods (supplementary Table 1) in order to standardize plasma n-3 PUFA, NO, and H_2_S status. Individuals were also asked to avoid alcohol or strenuous exercise in this 3-day period. The evening before their clinical visits, subjects were requested to consume a standard low-fat meal (<10 g of fat), which was provided. Adherence to the restrictions was assessed at each visit by questionnaire and a 24 h dietary recall.

On the clinical visit, baseline (0 h) vascular and BP measures were taken along with a fasting (>10 h) blood sample. Following the consumption of the test meal, which participants were requested to consume within a 5 min period, the vascular and BP measures and blood sampling were repeated at 4 h. The 4 h time point was selected to coincide with the predicted peak plasma lipemia and EPA and DHA plasma concentrations following lipid consumption (7).

### Meal design

Test meals were isocaloric [3,128 kJ; 9.5% of energy as protein, 51.0% of energy as fat (42.4 g total fat), and 39.5% of energy as carbohydrate] and matched for taste, smell, and appearance. They were composed of a high-fat chocolate-flavored milkshake containing the test fats and a peppermint extract to mask the fish oils, accompanied by toasted white bread (73 g) and a low fruit content jam (30 g). In the control meal, the milkshake was composed of 40 g of a 4:1 palm oil/soybean oil mixture, skimmed milk (150 g), chocolate-flavored powder (15 g), skimmed milk powder (15 g), and 2 g of peppermint oil extract. In the ERO- and DRO-test meals, 6.94 g and 8.33 g of the palm oil/soybean oil mixture was replaced by ERO or DRO (Epax^®^, FMC Health and Nutrition, Sandvika, Norway; see supplementary Table 2 for full fatty acid profile) to provide 4.16 g of EPA or DHA, respectively.

### Measures of BP and vascular function

All vascular measures were taken in a temperature-controlled room following a 15 min rest period. BP was taken using an Omron 750 CP-II device (Omron, Milton Keynes, United Kingdom) in a supine position. Arterial stiffness and endothelial dependent reactive hyperemia were assessed using PWV and the EndoPAT RHI, respectively. AIx, a measure of the enhancement of central aortic pressure by a reflected pulse wave, which is dependent on the vascular tone of resistance arteries, was also undertaken.

RHI was measured by arterial tonometry using an EndoPAT device (Itamar Medical Ltd., Caesarea, Israel) as previously described (18). Measures were taken for 5 min at rest, 5 min during brachial artery occlusion, and another 5 min subsequent to occlusion release. RHI was calculated by expressing the mean amplitude of peripheral arterial tone postocclusion relative to those at baseline. Nonocclusion effects were corrected for by accounting for changes in tone in the control arm probes. The RHI measure was also taken a second time at the postprandial (4 h) time point in response to 400 mg of sublingual nitroglycerine (GTN) in order to assess an impact of treatment on NO independent vasodilation.

A Vicorder device (Skidmore Medical, Bristol, United Kingdom) was used for the measurement of the PWV and AIx as described previously (19, 20). In brief, measures of carotid-femoral PWV were taken via placement of a BP cuff at the level of the carotid and femoral artery. The distance between the two anatomical sites was measured in each individual in order to allow calculation of pulse wave propagation speed in meters per second. For AIx, the Vicorder cuff was positioned over the brachial artery and AIx established by analysis of the BP wave form. Specifically, AIx was calculated by expressing augmentation pressure (the difference between BP during occurrence of the anacrotic notch and maximum SBP) as a percentage of total pulse pressure.

All BP and vascular measures were taken in triplicate apart from the RHI and RHI*GTN measures, which due to the basis of the methodology and the recovery time needed to return to premeasurement values, were measured only once.

### Serum/plasma lipids, glucose, nitrite, and H_2_S

Blood samples for lipid and glucose analyses were left to sit at room temperature for 30 min and centrifuged at 2,100 *g* at 4°C for 10 min. Serum samples were stored at −80°C until analysis. Serum triacylglycerol (TAG), TC, HDL-C, NEFA, and glucose were measured on an ILab650 automated analyzer (Instrumentation Laboratory, Warrington, United Kingdom) with commercially available kits (Instrumentation Laboratory).

Blood samples for nitrite and H_2_S quantification were drawn into ice-cold lithium heparin Vacutainers® and centrifuged immediately at 2,100 *g* at 4°C for 10 min. The obtained plasma samples were snap frozen and immediately stored at −80°C until analysis. Plasma nitrite analysis was conducted by reduction of nitrite to NO with chemiluminescent detection of NO undertaken via reaction with O_3_^−^ as previously described (21). H_2_S was measured by reverse-phase HPLC coupled with fluorescence detection after derivatization with monobromobimane, following methodology detailed by Shen et al. (22).

### Plasma fatty acid and oxylipin analysis

For fatty acid analysis, total lipids (TLs) were extracted and analyzed as described previously (23). In brief, TLs were extracted from 500 µl of plasma using chloroform-methanol (2:1, v/v). Fatty acid methyl esters (FAMEs) of the TL fraction were obtained by acid-catalyzed transmethylation, using nonadecanoic acid (19:0) (5% of the TL analyzed), as an internal standard. FAMEs were then purified by TLC and analyzed by gas-liquid chromatography.

Oxylipin analysis of plasma samples was carried out at the Institute for Vascular Signaling (Frankfurt, Germany). Briefly, samples were spiked with the corresponding deuterated internal standards (Cayman Europe, Tallinn, Estonia), and lipid epoxide and diol derivatives were extracted twice into ethyl acetate (0.5 ml) and quantified using a Sciex API4000 mass spectrometer operating in the multiple reaction monitoring mode as previously described (24, 25).

### Statistical analysis

Unless otherwise stated, data are presented as means ± SEM. Sample size calculations were undertaken with a power of 0.80 to detect clinically relevant changes at the 5% significance level for EndoPAT, PWV, and AIx. The primary outcome for the trial was the EndoPAT RHI index, with PWV and AIx as secondary outcomes. For EndoPAT, given an SD of 0.4 (26), a clinically relevant difference of 0.25 between treatments would be detectable in 21 subjects per treatment group. For PWV, given an SD of 1.2 m/s (27), a clinically relevant difference of 1.5 m/s would be detectable with a sample size of 11 participants per treatment group. For AIx, a clinically relevant 4% decrease in AIx would be detectable in a population of 13 individuals, assuming a population SD of 5% in AIx. We aimed to recruit 26 participants. This number was chosen to allow for a potential dropout rate of 15% and to provide a minimum of 22 participants completing the study.

Statistical analysis was undertaken using the SPSS statistical software (version 22; Chicago, IL) with *P* < 0.05 taken as being significant. Prior to analysis, all data sets were checked for normality via visual inspection of normal Q-Q plots. Outliers were identified by analysis of normal Q-Q plots and variance in values greater than ± 2 SD.

Two-factor repeated measures ANOVA was used to determine the independent and interactive impact of time and treatment on the outcome measures. Assumptions of sphericity were assessed via Mauchly’s test of sphericity. In cases where the assumptions of sphericity were not met, a Greenhouse-Geisser correction was applied. When significance was reached, subsequent post hoc analysis was undertaken to assess the significance of treatment group effect on the changes from baseline (4–0 h) using repeated measures ANOVA and Bonferroni adjustment. Pearson’s correlation analysis was used to establish the association between AIx responses and oxylipin levels.

## RESULTS

**Fig. 1.**
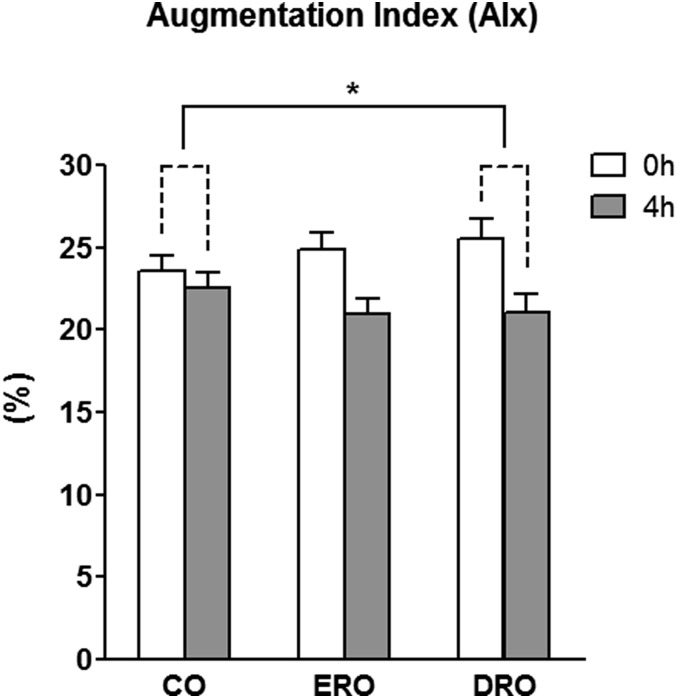
AIx (%) at baseline and in response to treatment. Data are presented as mean ± SEM (n = 26). Two-factor repeated measures ANOVA. *P*, time < 0.010; *P*, time × treatment = 0.005. The asterisk indicates significantly different (*P* = 0.047) change scores for DRO when compared with CO.

Twenty-six individuals with a mean age of 45 ± 5 years and BMI of 27.4 ± 3.3 kg/m^2^ completed all three arms of the trial (**Table 1**). The DHA-rich or EPA-rich interventions had no significant effect on BP, PWV, RHI, or RHI in response to sublingual GTN administration (**Table 2**). For AIx, there was a statistically significant time (0 h vs. 4 h) effect (*P* < 0.010) and time × treatment interaction (*P* = 0.005) (**Fig. 1**), and post hoc analysis revealed significant (13.3%, *P =* 0.047) and borderline significant (11.3%, *P =* 0.06) change scores for the DRO and the ERO meal compared with control. No time × treatment interactions were evident for TAG, NEFA, or glucose (*P* = 0.36, 0.90, and 0.49, respectively). However, time effects were observed with 40% (*P* < 0.001) and 15% (*P* = 0.02) higher TAG and NEFA, respectively, and 9% lower glucose (*P* < 0.001) levels at 4 h for all treatment groups combined (**Table 3**). No impact of time or treatment was evident for nitrite or H_2_S concentrations.

**TABLE 1.** Study population characteristics (n = 26)

Characteristic	Value
Age (years)	45 ± 5 (36–54)
Weight (kg)	87.0 ± 11.5 (73.5–129.4)
BMI (kg/m^2^)	27.4 ± 3.3 (20.4–39.9)
Waist circumference (cm)	95.5 ± 10.4 (76.8–128.0)
TC (mM)	5.8 ± 0.9 (3.8–7.3)
HDL-C (mM)	1.4 ± 0.3 (0.86–2.32)
SBP (mmHg)	136 ± 10 (101–150)
DBP (mmHg)	86 ± 7 (67–104)

Data are presented as mean ± SEM with the range of values in brackets.

**TABLE 2.** Vascular measurements at baseline (0 h) and in response to treatment (4 h)

	CO Meal		ERO Meal		DRO Meal			*P*, Time × Treatment*^a^*
	0 h	4 h	0 h	4 h	0 h	4 h	*P*, Time*^a^*	
DBP (mmHg)	77 ± 1	74 ± 2	77 ± 2	76 ± 2	75 ± 1	76 ± 1	0.25	0.15
SBP (mmHg)	125 ± 3	125 ± 2	127 ± 2	128 ± 2	125 ± 2	124 ± 1	0.93	0.38
RHI	2.4 ± 0.1	2.4 ± 0.1	2.4 ± 0.1	2.5 ± 0.1	2.4 ± 0.1	2.4 ± 0.1	0.75	0.92
RHI*GTN	—	1.7 ± 0.1	—	1.5 ± 0.1	—	1.6 ± 0.1	—	0.59
PWV (m/s)	8.5 ± 0.2	8.3 ± 0.2	8.3 ± 0.1	8.4 ± 0.2	8.2 ± 0.2	8.2 ± 0.2	0.85	0.32

RHI*GTN, RHI after 400 mg of sublingual GTN. Data are presented as mean ± SEM (n = 26).

a Two-factor repeated measures ANOVA.

**TABLE 3.** Biochemical measures at baseline and in response to treatment

	CO Meal		ERO Meal		DRO Meal			*P*, Time × Treatment*^a^*
	0 h	4 h	0 h	4 h	0 h	4 h	*P*, Time*^a^*	
Triacylglycerol (mM)	1.7 ± 0.1	2.5 ± 0.2	1.6 ± 0.1	2.3 ± 0.2	1.6 ± 0.1	2.2 ± 0.1	<0.001	0.36
NEFA (μM)	359 ± 24	424 ± 36	335 ± 32	384 ± 35	351 ± 31	398 ± 33	0.020	0.90
Glucose (mM)	5.2 ± 0.1	4.7 ± 0.1	5.2 ± 0.1	4.7 ± 0.1	5.2 ± 0.1	4.9 ± 0.1	<0.001	0.49
Nitrite (mM)	88.7 ± 8.6	80.1 ± 7.2	84.3 ± 6.2	74.3 ± 6.5	78.4 ± 6.5	81.9 ± 0.1	0.37	0.46
H_2_S (mM)	348.3 ± 59.3	386.6 ± 62.0	369.2 ± 59.6	341.7 ± 39.4	377.5 ± 63.6	386.4 ± 61.1	0.76	0.35

Data are presented as mean ± SEM (n = 26).

a Two-factor repeated measures ANOVA.

The ERO and DRO meals increased plasma EPA and DHA by 158% (*P* = 0.03) and 88% (*P* = 0.05), respectively. Post hoc analysis showed no significant change in docosapentaenoic acid (DPA) in response to any of the meals after a Bonferroni correction was applied (**Table 4**). A total of 7 EPA and DHA hydroxy, epoxide, and diols metabolites were quantified (Table 4). The ERO meal significantly increased plasma concentrations of 15S-HEPE, 14,15-EpETE, 17,18-EpETE, 14,15-DiHETE, and 17,18-DiHETE with the DRO also increasing concentrations of the EPA-derived metabolites 14,15-DiHETE, 17,18-EpETE, 17,18-DiHETE, and the DHA-derived metabolite 19,20-DiHDPA. No changes were evident following consumption of the control meal.

**TABLE 4.** Plasma LC n-3 PUFA concentrations along with select hydroxy, epoxide, and diol metabolites measured at baseline and in response to treatment (4 h)

	Control Meal		ERO Meal		DRO Meal			*P,* Time × Treatment*^a^*
	0 h	4 h	0 h	4 h	0 h	4 h	*P,* Time*^a^*	
Fatty acids (mg/ml)								
EPA	3.0 ± 0.1	3.5 ± 0.1	3.3 ± 0.1	8.5 ± 0.3*^b^*	3.7 ± 0.1	5.1 ± 0.1	<0.001	0.49
DPA	0.9 ± 0.1	1.0 ± 0.1	1.1 ± 0.1	0.7 ± 0.1	0.8 ± 0.1	0.9 ± 0.1	0.86	0.02
DHA	6.6 ± 0.2	6.4 ± 0.1	6.7 ± 0.2	7.5 ± 0.2	6.4 ± 0.2	12.0 ± 0.3*^b^*	0.007	0.006
Oxylipins (ng/ml)								
Hydroxy metabolites								
15S-HEPE	0.2 ± 0.1	0.1 ± 0.0	0.8 ± 0.5	2.6 ± 0.7*^b^*	0.4 ± 0.2	0.9 ± 0.3	0.05	0.03
18S-HEPE	0.5 ± 0.2	0.2 ± 0.1	1.8 ± 1.0	6.5 ± 2.3	0.5 ± 0.3	3.8 ± 1.6	0.02	0.07
Epoxide metabolites								
14,15-EpETE	0.6 ± 0.3	0.0 ± 0.0	0.4 ± 0.3	2.9 ± 0.8*^b^*	0.4 ± 0.2	0.5 ± 0.1	0.06	0.001
17,18-EpETE	0.9 ± 0.5	0.4 ± 0.0	0.6 ± 0.2	9.9 ± 1.8*^b^*	0.8 ± 0.3	2.6 ± 0.4*^b^*	<0.001	<0.001
Diol metabolites								
14,15-DiHETE	1.5 ± 0.1	1.5 ± 0.0	1.7 ± 0.1	3.2 ± 0.3*^b^*	1.6 ± 0.0	2.2 ± 0.1*^b^*	<0.001	<0.001
17,18-DiHETE	3.0 ± 0.5	2.6 ± 0.2	2.9 ± 0.3	9.7 ± 1.0*^b^*	2.8 ± 0.2	6.0 ± 0.4*^b^*	<0.001	<0.001
19,20-DiHDPA	0.9 ± 0.1	1.1 ± 0.1	0.8 ± 0.1	1.3 ± 0.1	0.9 ± 0.1	2.1 ± 0.2*^b^*	<0.001	<0.001

DiHDPA, dihydroxydocosapentaenoic acid; DiHETE, dihydroxyeicosatetraenoic acid; EpETE, epoxyeicosatetraenoic acid; HEPE, hydroxyeicosapentaenoic. Data are presented as mean ± SEM (n = 26).

a Two-factor repeated measures ANOVA.

b Indicates a significant difference in change from baseline when compared with control.

14,15-DiHETE, 17,18-DiHETE, and 19,20-DiHDPA were significantly negatively correlated with AIx (*r* = −0.215, *P* = 0.007; *r* = −0.223, *P* = 0.005; and *r* = −0.199, *P* = 0.013, respectively) (supplementary Table 5) with the AIx absolute response to DHA correlated with baseline AIx values (*r =* −0.413, *P* = 0.036).

## DISCUSSION

Arterial stiffness and vasodilation of the conduit arteries are important determinants of SBP, left ventricular hypertrophy, and overall CVD risk and are highly prognostic of future cardiovascular events (28, 29). Specifically AIx, a measure of pulse wave reflections influenced by vascular smooth muscle tone, affects central BP, with a reported hazard ratio of 1.68 [95% confidence interval (CI), 1.02–2.76] for all-cause mortality and 1.60 (95% CI, 1.07–2.39) for combined CVD end points for men in the highest versus lowest AIx tertile (30). In line with our recruitment of participants at above-average CVD risk, mean baseline AIx values of 24.6% were ∼30–50% higher than those observed in RCTs that included healthy males (31, 32) or in the lowest male CVD risk tertile in the Copenhagen City Heart Study (CCHS) (33) and were within the highest risk tertile for CCHS (33). Our main finding is an overall treatment effect on AIx, with the modest differences in responses between DHA and EPA intervention relative to control, although statistically different, unlikely to be of clinical significance. If sustained, the observed DHA-mediated 13.3% reduction would equate to a decrease in 10-year CVD risk from 3.3% to 2.8% in this population, using associations generated by the European Society of Cardiology (34). Furthermore EPA- and DHA-derived epoxides and diols were shown to be highly modifiable in the postprandial state and likely to contribute to the observed improved vascular function.

While to the best of our knowledge no previous RCT has compared the impact of EPA versus DHA on AIx, a limited number of studies have reported a positive impact of chronic and acute combined EPA + DHA supplementation on this measure of arterial stiffness (9, 10, 35, 36). Using a comparable LC n-3 PUFA exposure (4.7 g EPA + DHA; EPA/DHA, 0.67), Chong et al. (9) reported that the inclusion of fish oil in a test meal reduced AIx in the 1.5–4 h postprandial period in healthy adults, with an improved stiffness index only evident in male participants. Purcell et al. (10) observed significant reductions in AIx in healthy men after fish oil (5 g EPA + DHA; EPA/DHA, 1.6) and algal oil (5 g DHA only) administration, during a 6 h postprandial assessment, which were most evident at 2 h and of comparable size effect to those observed in the current RCT. However, they did not include an EPA-only meal, which would allow direct comparison of the vasoactivity of EPA versus DHA on AIx and other measures of vascular function (10).

In the current study, no effect of the EPA or DHA meals on PWV or RHI was observed. PWV is a proximal measure of gross arterial stiffness determined by the speed at which the pulse wave travels through the arterial tree, with an increased speed indicating increased stiffness and overall vascular dysfunction (28). In a meta-analysis of 17 studies, an increased PWV was associated with an increased risk of total cardiovascular events, cardiovascular mortality, and all-cause mortality [RR (95% CI) of 2.26 (1.89–2.70), 2.02 (1.68–2.42), and 1.90 (1.61–2.70), respectively] (37). The observed lack of impact of treatment on postprandial PWV in the present study corroborates previous observations showing that PWV is not acutely modified in response to altered fatty acid composition (38, 39).

Peripheral arterial tonometry RHI as assessed by the proprietary EndoPAT device was used as an observer-independent, high-throughput technique to assess endothelium-dependent vasodilation (EDV) (40). Since this study was initiated, concerns have been expressed regarding the sensitivity of the EndoPAT technique, in particular its ability to assess (subtle changes in) endothelial function in health individuals or at the early stages of vascular dysfunction (41, 42). Future trials should use more sensitive techniques, such as flow-mediated dilatation, in order to assess the impact of EPA versus DHA on postprandial EDV. The prognostic value of a compromised flow-induced vasodilation (43), the observation of improved response to flow following chronic EPA + DHA supplementation (3) and of a differential and stronger impact of DHA relative to EPA using a less widely used and indirect measure of EDV, namely systemic vascular resistance as measured by the Finometer finger arterial BP monitor (11), provides strong justification for such research to be conducted.

Much of the mechanistic focus centered around the modulation of vascular function by EPA and DHA has been on NO bioavailability and endothelial NO synthase expression and phosphorylation status (44). There has been a degree of inconsistency with regard to the capacity of LC n-3 PUFA to modify NO production postprandially, with some studies indicating an effect (8), whereas others have observed no changes (10). As NO is a labile compound, its status is often estimated via the concentrations of its oxidation products, most notably nitrite and nitrate, with nitrite thought to be the most reflective of changes in NO concentration (45). In agreement with previous investigations utilizing this method, there was no evidence of postprandial EPA- or DHA-mediated changes in plasma levels of nitrite or total NO metabolites (10).

H_2_S has recently emerged as a novel endothelium-derived regulator of vascular function (16, 46). Although the impact of EPA and DHA on H_2_S status in the vasculature is almost completely unknown, a recent study reported an activation of cystathionine-γ-lyase, the main H_2_S biosynthesis enzyme, in response to DHA-rich tuna-oil supplementation in the lung tissue of Sprague-Dawley rats (47). However, no EPA- or DHA-mediated differences were evident in the current RCT, and it is unlikely that H_2_S contributes to reduced arterial stiffness associated with acute DHA consumption.

Finally, our study was novel in its investigation of the impact of EPA versus DHA on the plasma concentration of selected vasoactive cytochrome P450 (CYP) enzyme-derived EPA and DHA metabolites (oxylipins), and for the first time concurrent assessment of the impact of LC n-3 PUFA on the vasoactive oxylipin profile and vascular responses in humans was conducted. Previous in vitro, ex vivo, and animal investigations have shown that the LC n-3 PUFA epoxides are particularly potent, and that the DHA epoxides account for ∼75% of DHA’s capacity to illicit vasodilation in in vitro models (48). Furthermore, the DHA epoxides have emerged as being more vasoactive than their EPA counterparts (15, 48, 49). In 2010, Shearer et al. (50) reported for the first time the presence of EPA- and DHA-derived epoxide and diols in human plasma and their modulation by EPA + DHA supplementation, which has been subsequently corroborated (51, 52). Consistent with the only previous acute assessment (52), which used a supraphysiological DHA-rich fish oil dose (26 g EPA + DHA; EPA/DHA, 0.12), it can be confirmed that the hydroxy, epoxide, and diol oxylipins are highly modifiable in the postprandial state with several fold higher concentration evident at 4 h postconsumption of test meals containing 4.16 g of EPA or DHA.

The DHA-derived diol 19,20-epoxydocosapentaenoic acid (19,20-EpDPE) has emerged as a potentially potent vasomodulator with direct effects on vascular smooth muscle tone (48). A limitation of this study is that we were not able to detect 19,20-EpDPE, which fell below the limit of detection. Results from the human serum metabolome project suggest that the molar ratio of 19,20-EpDPE to 19,20-DiHDPA is in the range of 1 in 8 (53), which places plasma concentrations of 19,20-EpDPE in the low picomolar range. However, we did observe a highly significant 1.3-fold increase in the daughter 19,20-DiHDPA diol in response to DHA consumption, with 19,20-DiHDPA concentration negatively correlated with AIx responses. Given that 19,20-DiHDPA is directly and exclusively derived from 19,20-EpDPE via the activity of the soluble epoxide hydrolase, the higher postprandial 19,20-DiHDPA levels are likely reflective of increases in 19,20-EpDPE, which is proposed to have contributed to the vascular impact of DHA versus EPA.

However, we cannot preclude the possibility that EPA-derived oxylipins also contributed to the vascular response following DRO intervention. The strong trend toward an increase in plasma EPA following DRO intervention, was likely due to some EPA being provided as part of the DRO intervention or the known tissue retroconversion of DHA to EPA (54). These sources provided sufficient amounts for the CYP epoxygenases that generally prefer EPA over DHA and arachidonic acid (55), with the DRO meal resulting in an increase in three EPA-derived oxylipin metabolites, with 14,15-DiHETE and 17,18-DiHETE correlated with AIx. In addition to the CYP-derived oxylipins, prostanoids and lipoxygenase-derived resolvins may contribute to the vascular response to n-3 PUFA intervention. Although potentially important in the vascular wall, this latter group of compounds has proved difficult to quantify in the human circulation.

Therefore, following DHA intervention changes in the tissues status of both DHA and EPA and their associated oxylipins and other metabolites may act in a complementary and additive fashion (56) to mediate the impact on vascular function.

The strengths of the current study were its crossover design, its inclusion of a range of measures of vascular function, with simultaneous analysis of vascular function and potential lipid- and nonlipid-derived mediators in the circulation at the clinical assessment time points. A limitation is the likely lack of sensitivity of the EndoPAT technique used to assess the impact of treatment on EDV.

In summary, the results of this study show that DHA improves postprandial vascular function, with an effect size that would translate into a meaningful reduction in CVD risk, with a strong trend also evident following EPA intervention. The targeted lipidomic profiling indicates that postprandial changes in the LC n-3 PUFA epoxides and diol occur and are likely to play a mechanistic role as effector molecules of the vascular responses. Further research is needed to fully establish the individual impact of EPA versus DHA on clinical end point and disease biomarkers and to gain insight into the bioactive lipids modulating the effects. Ultimately, such information would allow the refinement of current EPA and DHA recommendations and the development of fish oil blends (with defined EPA and DHA content) to suit phenotype and would inform the design of much needed nonmarine sources of these fatty acids.

## Supplementary Material

Supplemental Data
