# Supplementary material for: Differential effects of EPA versus DHA on postprandial vascular function and the plasma oxylipin profile in men
Source: J Lipid Res. 2016 Sep;57(9):1720–7. doi: 10.1194/jlr.M067801 (PMC5003154; doi:10.1194/jlr.M067801)
Supplement: Supplemental Data [file supp_57_9_1720__index.html]

Differential effects of EPA vs. DHA on postprandial vascular function and the plasma oxylipin profile in men — Differential effects of EPA vs. DHA on postprandial vascular function and the plasma oxylipin profile in men — Differential effects of EPA versus DHA on postprandial vascular function and the plasma oxylipin profile in men — Supplemental Data 

# Differential effects of EPA versus DHA on postprandial vascular function and the plasma oxylipin profile in men

## Supplemental Data

- Supplemental Tables 1 to 5 (.pdf, 284 KB) - Table S1. List of food to be avoided for the three days prior to clinical visits. Table S2. Estimated fatty acid composition (%) of meal as derived from the manufacturers data. Table S3. Plasma fatty acid concentrations (mg/ml) at baseline and in response to treatment. Table S4. Supporting data for Figure 1: Augmentation index (%) at baseline and in response to treatment. Table S5. Bivariate correlations between oxylipins concentrations and Augmentation Index (AIx).
