# Supplementary material for: Differential effects of EPA versus DHA on postprandial vascular function and the plasma oxylipin profile in men
Source: J Lipid Res. 2016 Sep;57(9):1720–7. doi: 10.1194/jlr.M067801 (PMC5003154; doi:10.1194/jlr.M067801)
Supplement: Supplemental Data [file 10.1194_M067801_jlr.M067801-1.pdf]

Online Supplemental Data: Tables

Differential effects of EPAvsDHA on postprandial vascular function and the plasma oxylipin profile in at-risk men

Seán McManus[1†], Noemi Tejera[1†], Khader Awwad[2], David Vauzour[1,3], Neil Rigby[3], Ingrid Fleming[2], Aedin Cassidy[1], and Anne Marie Minihane[1*]

[1] Department of Nutrition and Preventive Medicine, Norwich Medical School, University of East Anglia, United Kingdom; [2] Institute for Vascular Signalling, Centre for Molecular Medicine, Goethe-University, Frankfurt, Germany; [3] Institute of Food Research, Norwich Research Park, Norwich NR47UA, United Kingdom.

[†] joint first author

See the following pages for Supplemental Table S1, Table S2, Table S3, Table S4 and Table S5.

Table S1. List of food to be avoided for the three days prior to clinical visits.

**Food rich in omega 3 Fatty acids**
Oily fish
n-3 PUFA enriched eggs
Vegetable oil spreads
**Nitrate- and nitrite-rich foods**
Leafy green vegetables
Broccoli
Beetroot
Radishes
Carrots
Sausages
Processed or cured meats
Mineral water containing >0.1mg/L nitrate
**Sulphite- and sulphate-rich foods**
Onions
Garlic
Wine
Dried fruits or meats
Nut/trail mixes
Shredded coconut
Brussel sprouts
Cabbage
Broccoli
Red peppers

Table S2. Estimated fatty acid composition (%) of meal as derived from the manufacturers data.

Fatty acid	CO-meal	ERO-meal	DRO-meal
**Palmitic acid, C16:0**	32.15	27.36	26.49
**Palmitoleic acid, C16:1**	n/a	0.46	0.76
**Stearic acid, C18:0**	3.62	3.02	2.90
**Oleic acid, C18:1n-9**	40.47	35.32	34.87
**Linoleic acid, C18:2n-6**	22.27	18.40	17.63
**α-Linolenic acid, C18:3n-3**	1.35	1.58	1.84
**Stearidonic acid, C18:4n-3**	n/a	0.46	0.76
**Arachidic acid, C20:0**	0.13	0.11	0.11
**Gondoic acid, C20:1n-9**	n/a	0.46	0.55
**Arachidonic acid, C20:4n-6**	n/a	0.46	0.76
**Eicosapentaenoic acid, C20:5 n-3**	n/a	9.82	1.96
**Heneicosapentaenoic acid, C21:5n-3**	n/a	0.46	0.76
**Docosapentaenoic acid, C22:5n-3**	n/a	0.46	0.76
**Docosahexaenoic acid, C22:6 n-3**	n/a	1.64	9.82

CO: Control oil; ERO: EPA rich oil; DRO: DHA rich oil; n/a: not applicable.

Table S3. Plasma fatty acid concentrations (mg/ml) at baseline and in response to treatment.

Fatty acid	Control meal		ERO-meal		DRO-meal	
	(0h)	(4h)	(0h)	(4h)	(0h)	(4h)
**Palmitic acid, C16:0**	87.0 ± 14.9	89.9 ± 7.8	90.0 ± 8.4	90.6 ± 7.7	86.5 ± 10.0	91.7 ± 7.9
**Palmitoleic acid, C16:1**	8.7 ± 2.1	7.1 ± 0.6	8.7 ± 0.9	7.8 ± 0.9	7.8 ± 1.0	7.6 ± 0.8
**Stearic acid, C18:0**	28.3 ± 4.8	29.3 ± 2.7	28.5 ± 2.8	27.3 ± 2.3	27.9 ± 3.4	28.1 ± 2.4
**Oleic and Vaccenic acids, C18:1[1]**	95.0 ± 4.8	98.3 ± 6.9	95.7 ± 10.0	95.3 ± 8.7	95.2 ± 10.9	102.6 ± 9.0
**Linoleic acid, C18:2 n-6**	105.9 ± 19.4	103.5 ± 8.3	102.0 ± 10.6	99.5 ± 8.8	95.3 ± 7.9	100.7 ± 8.3
**alpha Linolenic acid, C18:3 n-3**	2.5 ± 0.4	2.9 ± 0.2	2.2 ± 0.3	3.0 ± 0.3	2.8 ± 0.6	3.1 ± 0.3
**gamma Linolenic acid, C18:3n6**	1.6 ± 0.2	1.7 ± 0.2	1.7 ± 0.2	1.8 ± 0.2	2.2 ± 0.5	1.8 ± 0.2
**Dihomo-gamma-linolenic acid, C20:3 n-6**	6.4 ± 1.3	5.8 ± 0.6	6.4 ± 0.7	6.0 ± 0.5	5.9 ± 0.7	5.9 ± 0.5
**Arachidonic acid, C20:4 n-6**	24.8 ± 3.5	24.2 ± 2.2	25.5 ± 3.0	23.7 ± 2.3	23.9 ± 3.6	24.1 ± 2.4
**Eicosapentaenoic acid, C20:5 n-3[234]**	3.0 ± 0.4	3.5 ± 0.6	3.3 ± 0.4	8.5 ± 1.5*	3.7 ± 0.7	5.1 ± 0.6
**Docosapentaenoic acid, C22:5 n-3[4]**	0.9 ± 0.1	1.0 ± 0.2	1.1 ± 0.2	0.7 ± 0.1	0.8 ± 0.1	0.9 ± 0.1
**Docosahexaenoic acid, C22:6 n-3[234]**	6.6 ± 1.3	6.4 ± 0.7	6.7 ± 0.8	7.5 ± 0.8	6.4 ± 0.9	12.0 ± 1.3*

Data presented as mean ± SEM. CO: Control oil; ERO: EPA rich oil; DRO: DHA rich oil. [1]Contains n-9 (Oleic acid) and n-7 (Vaccenic acid) isomers, [2]Indicates significant effect for time by Repeated Measures ANOVA, [3]Indicates significant effect for treatment by Repeated Measures ANOVA, [4]Indicates significant effect for time*treatment effect by Repeated Measures ANOVA. *Indicates a significant difference in change from baseline when compared to control.

Table S4. Supporting data for Figure 1: Augmentation index (%) at baseline and in response to treatment.

**AIx (%)**	**Control Meal**		**ERO-meal**		**DRO-meal**	
	(0h)	(4h)	(0h)	(4h)	(0h)	(4h)
	23.6 ± 0.9	22.5 ± 0.9	24.9 ± 1.0	21.0 ± 0.9	25.5 ± 1.2	21.1 ± 1.1*

Data are presented as mean ± SEM (n = 26). CO: Control oil; ERO: EPA rich oil; DRO: DHA rich oil. Two-factor repeated measures ANOVA. P, time < 0.010, P, time*treatment = 0.005. * indicates significantly different (P = 0.047) change scores for DRO when compared to CO.

Table S5. Bivariate correlations between oxylipins concentrations and Augmentation Index (AIx).

**Oxylipins**	Pearson correlation with AIx (*r*)	P-value
14, 15-EpETE	-0.010	0.902
14, 15-DiHETE	-0.215*	0.007
17, 18-EpETE	-0.047	0.558
17, 18-DiHETE	-0.223*	0.003
15S-HEPE	0.005	0.954
18S-HEPE	0.015	0.851
19, 20-DiHDPA	-0.199*	0.013

* Correlation is significant at the 0.05 level (2-tailed).
